# Biocatalytic CO_2_ Absorption and Structural Studies of Carbonic Anhydrase under Industrially-Relevant Conditions

**DOI:** 10.3390/ijms21082918

**Published:** 2020-04-22

**Authors:** Aline M. de Castro, Elisabete Ferreira, Carla Portugal, Luisa A. Neves, João G. Crespo

**Affiliations:** 1 LAQV-REQUIMTE, Departamento de Química, Faculdade de Ciências e Tecnologia, Universidade NOVA de Lisboa, 2829-516 Caparica, Portugal; cmp@fct.unl.pt (C.P.); lan11892@fct.unl.pt (L.A.N.); 2 Biotechnology Division, Research and Development Center, PETROBRAS, Av. Horácio Macedo, 950. Ilha do Fundão, Rio de Janeiro 21941-915, Brazil; 3 UCIBIO-REQUIMTE, Departamento de Química, Faculdade de Ciências e Tecnologia, Universidade NOVA de Lisboa, 2829-516 Caparica, Portugal; ep.ferreira@fct.unl.pt

**Keywords:** carbonic anhydrase, greenhouse gases, CO_2_ capture, circular dichroism, fluorescence

## Abstract

The unprecedently high CO_2_ levels in the atmosphere evoke the urgent need for development of technologies for mitigation of its emissions. Among the alternatives, the biocatalytic route has been claimed as one of the most promising. In the present work, the carbonic anhydrase from bovine erythrocytes (BCA) was employed as a model enzyme for structural studies in an aqueous phase at alkaline pH, which is typical of large-scale absorption processes under operation. Circular dichroism (CD) analysis revealed a high enzymatic stability at pH 10 with a prominent decrease of the melting temperature above this value. The CO_2_ absorption capacity of the aqueous solutions were assessed by online monitoring of pressure decay in a stainless-steel cell, which indicated a better performance at pH 10 with a kinetic rate increase of up to 43%, as compared to non-biocatalytic conditions. Even low enzyme concentrations (0.2 mg g^−1^) proved to be sufficient to improve the overall CO_2_ capture process performance. The enzyme-enhanced approach of CO_2_ capture presents a high potential and should be further studied.

## 1. Introduction

The impact on the planet and on society caused by the increasing emission of greenhouse gases to the atmosphere has raised the urgent need for development of technologies for mitigating gas emissions and their capture. Anthropogenic CO_2_ emissions can be controlled by implementing efficient processes of gas capture, which may include physical, chemical, and/or biochemical approaches [1,2,3]. Among them, the use of the metallo hydro-lyase enzyme named carbonic anhydrase (CA, EC 4.2.1.1) has been reported as one of the most promising paths [4]. 

CA catalyzes the reversible reaction that involves the hydration of CO_2_ to bicarbonate with concomitant release of protons (Reaction (R1)) and with an unprecedently high reaction rate (kcat ≈ 10^6^ s^−1^) [5]. This kinetic potential, as well as the ubiquitous occurrence of CAs in nature [6], entitle this enzyme as a good biocatalyst for industrial applications, such as CO_2_ capture from air, post-combustion, biogas, and natural gas streams [3,7]. From this group, the CA from bovine erythrocytes (BCA) has been one of the most studied due to its activity under a wide pH range and commercial availability [8,9], among other interesting properties.

(R1) $$CO_{2}+H_{2}O\;\overset{CA}{\Leftrightarrow }HCO_{3}^{-}+H^{+}$$

The biocatalytic CO_2_ capture mediated by CAs has been studied in a wide range of aqueous solvents, most likely in the presence of promoters, as amines (e.g., monoethanolamine-MEA, N-methyldiethanolamine-MDEA) [10] and salts, such as inorganic carbonates (K_2_CO_3_, Na_2_CO_3_) [11,12], and ionic liquids under high water activity (a_w_) conditions (e.g., 1-butyl-3-methyl-imidazolium bis(trifluoromethanesulfonyl)imide-[C_4_mim][Tf_2_N], at a_w_= 0.836) [13]. Therefore, the comprehension of the enzyme performance under different environmental conditions is important for the development of industrially-relevant processes of CO_2_ capture.

Due to its key role in living organisms, the previous structural investigations on BCA have focused on the elucidation of its properties under physiological conditions (pH below 7.5) to address queries related to life sciences [8,9,14,15]. Thus, to the best of our knowledge, there is a lack of studies and available data that relate BCA structural properties and its performance under industrially-relevant CO_2_ capture conditions (mostly alkaline pH conditions) [10,11,12]. 

In this study, the effect of relevant variables, such as pH and enzyme concentration, were assessed during CO_2_ capture in aqueous media. Changes on the BCA enzyme conformation, under environmental conditions typical of industrial CO_2_ capture, were observed using well established techniques such as circular dichroism (CD) and fluorescence emission. 

## 2. Results and Discussion

### 2.1. Characterization of Aqueous Media and BCA

First, the mineral water (MW) used in the work was characterized according to its ionic composition (Table 1). The electric conductivity and pH (before pH adjustment to specific test conditions) were determined to be 0.472 mS cm^−1^ and 8.77 mS cm^−1^, respectively. MW was used instead of deionized water due to the easier controllability of its pH and proximity of the original pH to the desired conditions of the tests. Under an alkaline environment, besides Reaction (R1), Reaction (R2) is also likely to occur, which favors the overall absorption performance [16].

(R2) $${\mathrm{CO}}_{2}{+\mathrm{OH}}^{-}\;\overset{\mathrm{CA}}{\Leftrightarrow }{\mathrm{HCO}}_{3}^{-}$$

**Figure 1:**
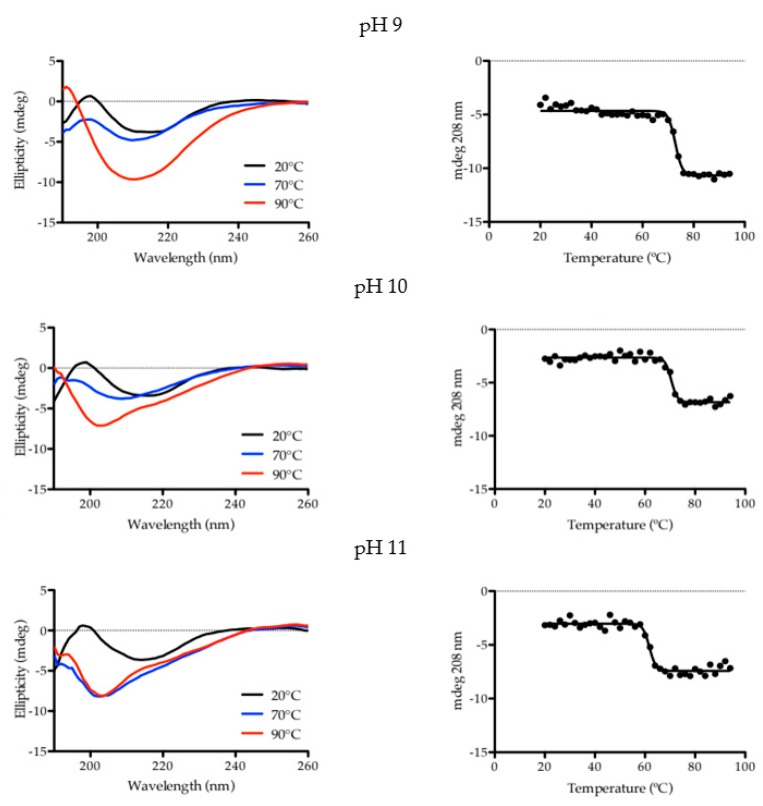
Thermal stability of BCA monitored by CD spectroscopy. **Left side**: CD spectra of the thermal unfolding of BCA under different pH conditions followed by circular dichroism (for conciseness, only initial, mid and final curves are shown). **Right side**: Melting curves of BCA under different pH conditions monitored by CD at 208 nm. The data were fitted using a two-state model.

BCA samples were observed under different conditions and techniques. CD analyses of the enzyme in aqueous solution were carried out to investigate the effect of pH in its thermal stability and overall stability over time. For each pH condition, 38 spectra containing 71 data points each, were acquired from 20 to 94 °C (with a 2 °C interval), and the signals at 208 nm were plotted as a function of temperature (Figure 1). It is clear to see that the enzyme melting temperature lowers as the pH becomes more alkaline. The melting temperature (T_m_) of BCA decreased 11 °C in total, from pH 9 to pH 11 (Table 2). The increase of the absolute signal at 208 nm suggests that BCA only partially unfolds and may have considerable residual alpha helical content in a “molten globule state”, which is a state found in other forms of carbonic anhydrase [17,18,19].

In all cases, the T_m_ values were found to be below typical temperatures adopted in large-scale stripping columns used in CO_2_ absorption plants (>75 °C) [15], which indicates that, in the event of designing an upscaled process, this enzyme must be kept in the absorption column, to operate at temperatures below its T_m_. This can be achieved through protein immobilization in supports, which can then be used in different absorption process operations, including bubble columns [20] and stirred reactors [21,22].

**Figure 2:**
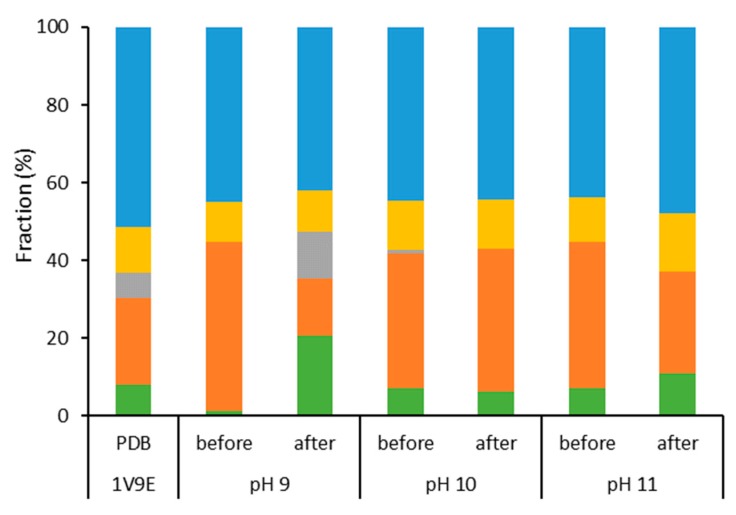
Secondary structure composition of BCA before and after a temperature ramp (20–94 °C) under different pH conditions in mineral water, as determined from Circular Dichroism analysis and treatment in the BeStSel base. Bars represent the fractions of α-helix (green), antiparallel β-sheet (orange), parallel β-sheet (grey), turn (yellow), and others (blue) in the structure. The protein concentration was 0.5 mg g^−1^.

After heating until 94 °C, the protein was analyzed again at 20 °C, and it is possible to observe that, at pH 9 and pH 11, the changes in the CD spectra caused by heating are maintained and, at pH 10, BCA seems to revert to a CD profile similar to the initial one (Figure S1). This correlates to the changes in the secondary structure (Figure 2) where it is possible to observe that, under pH 9 and pH 11, the BCA structure changes more significantly due to the decrease in the antiparallel β-sheet portion (in orange), and due to the increase in the α-helix (green) at pH 9. On the other hand, at pH 10, the structure seemed to be less damaged by the temperature ramp, with a slightly lower content of parallel β-sheet than the original protein (PDB reference). Altogether, these observations indicate that the changes in secondary structure are more permanent at pH 9 and pH 11. At pH 10, the denaturation of the enzyme is more reversible.

Dramatic changes in the BCA secondary structure were not expected in any condition tested in this study. As reported by Gudiksen [23], even at known denaturing conditions, such as in the presence of saturated guanidinium chloride solutions, BCA is not completely unfolded, and tends to form aggregates when in the partially unfolded state.

**Figure 3:**
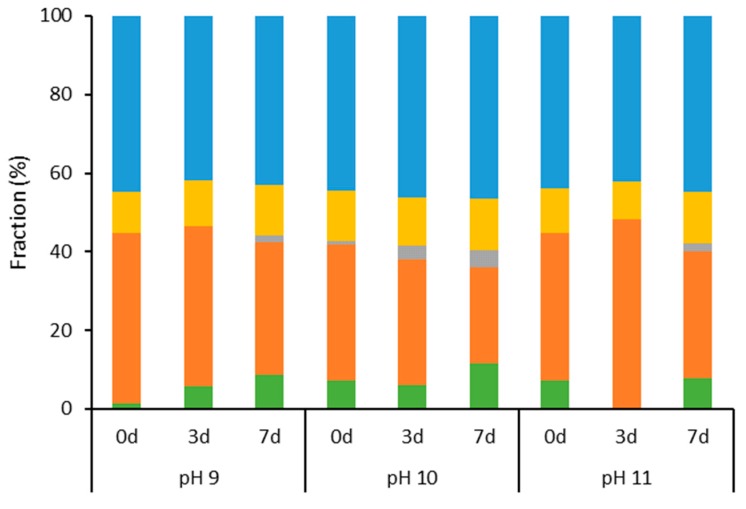
Secondary structure composition of BCA during incubation at 30 °C under different pH conditions in mineral water, as determined from Circular Dichroism analysis and treatment in the BeStSel base. Bars represent the fractions of α-helix (green), antiparallel β-sheet (orange), parallel β-sheet (grey), turn (yellow), and others (blue) in the structure. The protein concentration was 0.5 mg g^−1^.

The protein stability was also assessed during incubation at 30 °C, under the three pH conditions (Figure 3). In this set of data, only slight changes in the proportion of the fractions as well as in CD spectra (Figure S2) were observed in all cases.

The influence of pH on the structure of BCA, in the acidic range, was studied using the fluorescence-anisotropy technique by Bushmarina et al. [9]. The authors observed more prominent structural changes at pH 3.5–4.5, which was correlated with the mobility of tryptophan residues. These authors also concluded that the pH-induced unfolding of this enzyme leads to the formation of two partially-folded intermediates, which is a different behavior as compared to guanidinium chloride-induced unfolding.

**Figure 4:**
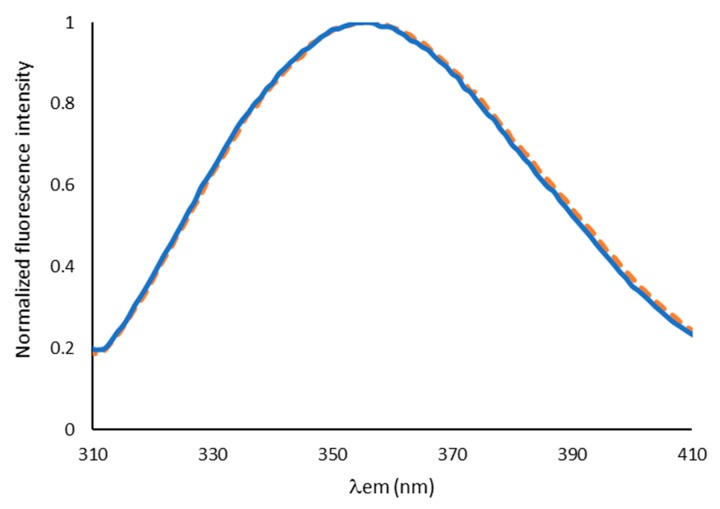
Fluorescence emission spectra of BCA in unbuffered (orange, dotted line) and buffered (blue, continuous line) mineral water. Analyses were carried out at circa 25 °C (room temperature) and protein concentration was 0.5 mg g^−1^.

A fluorescence analysis was also performed in the present work. In this case, the technique supported the observation of the protein in the presence and absence of a buffered environment, which revealed no significant changes, from the point of view of exposure of the aromatic residue of tryptophan (Figure 4). Only a subtle band shift (<1 nm) to lower emission wavelengths (blue shift) was observed in the presence of the salt. Although the spectral changes are not significant to support the existence of protein structural changes caused by the presence of the buffer, the blue shift would be compatible with a decrease of the overall exposure of tryptophans to the external media and, thus, to a higher protein folding.

### 2.2. CO_2_ Absorption Tests

**Figure 5:**
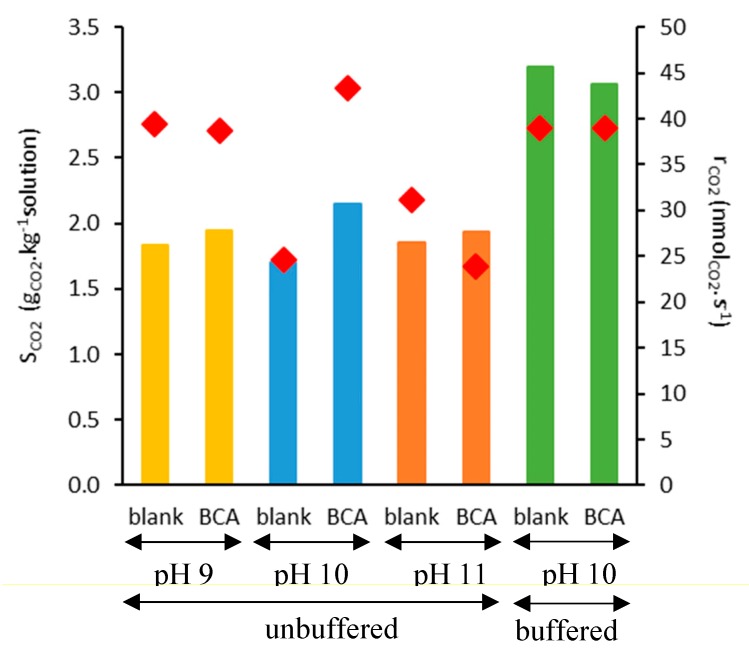
S_CO2_ (bars) and r_CO2_ (diamonds) results of CO_2_ sorption tests in mineral water in unbuffered conditions at pH 9, pH 10, and pH 11 and buffered condition at pH 10. Tests were carried out in duplicate with 2 g of sample at p_i_ = 1.5 bar, 30 °C, and 0.5 mg_protein_ g^−1^_solution_ (when used). The mean standard deviation is 4% for S_CO2_ data and 8% for r_CO2_ data.

The influence of pH on the BCA performance for CO_2_ absorption was kinetically investigated through online monitoring of pressure decay as a result of the gas (CO_2_) capture in the aqueous phase (Figure S3). As shown in Figure 5, the addition of BCA to each test condition had a low influence on the overall sorption coefficient (S_CO2_), determined at equilibrium. However, under unbuffered conditions, the presence of the enzyme speeds up the initial capture rate (r_CO2_) by 76% (at pH 10). No positive effect was observed at pH 9 as well as at pH 11. The best pH condition observed in the present study was within the range reported by Wojtasik et al. [24] (pH 10–10.3) during CO_2_ capture in a 30 wt% methyldiethanolamine (MDEA) solution. When considering a buffered environment, the observation was that S_CO2_ was increased by 35% in the presence of the enzyme, as compared to an unbuffered condition at the same initial pH. The enzyme, however, did not exert a positive influence for the buffered condition, as compared to the use of buffer in the absence of BCA (blank assay). This may be related to the possibility of CA action toward CO_2_ hydration products (reversibility of the reaction, as shown in Reactions (R1) and (R2)), so that the overall effect might have been neutralized in this condition.

The S_CO2_ and r_CO2_ values reported in this case can be increased in future tests by the adoption of the following conditions: (1) addition of an absorption promoter (e.g., amine, inorganic carbonate), (2) higher partial pressure in the absorption cell since it is well known that pressure exerts a strong effect in gas solubility in liquid phases [25]. Additionally, r_CO2_ may be improved by utilization of a mixing device in the absorption cell, as previously stated during the comparison of fixed and rotating packed beds for biocatalytic CO_2_ capture [24].

After the absorption tests at pH 9, 10, and 11, the pH and electric conductivity of solution were measured. The results shown in Table 3 indicate that, at pH 9, there was an increase in the conductivity, characterized by the incorporation of the CO_2_ as HCO_3_^−^ in the liquid phase, whereas, at a more alkaline pH (pH 11), the conductivity decreased, which suggests precipitation of salts, most likely in the form of carbonates (Table 1) [26]. The different behaviors observed by the addition of BCA at pH 10 and pH 11 are supported by the conclusions given by Li et al. [27] regarding the effects of CA on the calcium carbonate salts morphology (e.g., formation of calcite or vaterite crystals).

When assessing the influence of a buffered environment (at pH 10), there was an increase in the S_CO2_ values, but no advantage of using BCA under the view of kinetic performance, as compared to the biocatalytic, unbuffered condition. According to our preliminary analyses, the carbonate-bicarbonate buffer was shown to be a good choice for this reaction, as compared to other buffers, such as glycine-NaOH (50 mM, pH 10), which yielded a S_CO2_ of 2.3 g_CO2_ kg^−1^_solution_ and an r_CO2_ of 40.1 nmol_CO2_ s^−1^.

**Figure 6:**
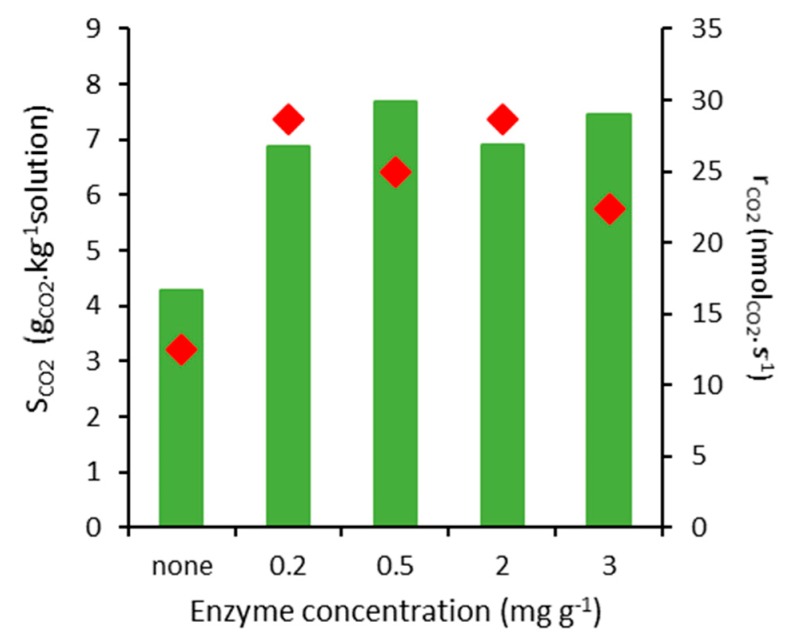
S_CO2_ (bars) and r_CO2_ (diamonds) results of CO_2_ sorption tests in mineral water in unbuffered conditions at pH 10. Tests were carried out in duplicate with 0.5 g of sample at initial p_i_ = 1.5 bar, 30 °C. The mean standard deviation is 4% for S_CO2_ data and 8% for r_CO2_ data.

Regarding the effect of enzyme concentration in the absorption performance, it is possible to observe from Figure 6 that low biocatalyst concentrations are sufficient to favour the absorption, and higher concentrations do not seem to improve the reaction yield. A similar pattern was observed during biocatalytic CO_2_ absorption in other alkaline conditions with a linear performance increase at low enzyme concentrations (<0.5 g L^−1^) and subsequent asymptotic deviation at higher concentrations [11,28,29]. When calculating the specific absorption as a function of the enzyme amount added (and excluding any contribution of water), it is clear that the most interesting condition is obtained with 0.2 mg g^−1^ of the enzyme (Table 4), which is attractive from an economic point of view.

## 3. Materials and Methods

### 3.1. Materials

Carbonic anhydrase from bovine erythrocytes (product code C2624, batch# SLBW5860, containing >3500 Wilbur-Anderson units/mg protein) and CO_2_ (99.998% purity) were purchased from Sigma-Aldrich (Algés, Portugal) and Praxair (Porto Maia, Portugal), respectively. Mineral water was purchased from a local market (Monchique brand, alkaline pH). Buffered conditions consisted of sodium carbonate-bicarbonate buffer 100 mM at pH 10.

### 3.2. Solution Characterization

Conductivity and salinity were determined in a Hach SensIon^+^ EC7 conductometer, whereas pH was recorded with a Crison pHmeter (model Basic 20).

The concentration of specific cations was determined by Induced coupled plasma—atomic emission spectroscopy (ICP-AES) (Horiba Jobin-Yvon, Quioto, Japan), model Ultima, equipped with 40.68 MHz generator, monocromator Czerny-Turner and autosampler AS500. The concentration of anions was obtained by High Performance Ion Chromatography (HPIC), in a Dionex ICS3000 (Sunnyvale, CA, USA), provided with Thermo Ionpac AS11-HC 250 × 4 mm + AG11HC columns. A solution of 30 mM NaOH at a flowrate of 1 mL/min, was used as eluent. The column temperature was 25 °C, the injection volume was 10 μL, and detection was carried out based on conductivity.

### 3.3. Enzyme Characterization

Fluorescence emission measurements were performed in a Spex Fluorolog 3 spectrofluorometer (Horiba) using an excitation wavelength (λ_ex_) of 295 nm and emission wavelength (λ_em_) ranging from 300–550 nm with excitation and emission slits of 5 nm. Full spectra were recorded in Datamax software.

Circular Dichroism (CD) analysis was carried out in a Chirascan qCD, from Applied Photophysics, provided with a Quantum Northwest TC125 temperature controller. The quartz cuvette path length was 0.2 mm. The temperature ramp spectra were acquired with 0.3 s per point, single scan, 1 nm of step and bandwidth, from 20 to 94 °C in a stepped ramp mode (step 2 °C, 60 s of stabilization). For determination of the T_m_, data was fitted using a two-state model in the Prism 5 software. The other CD spectra were acquired with 3 s per point, 3 scans, and 1 nm of step and bandwidth. For the determination of secondary structure content, raw CD data were treated in the BeStSel base [30,31].

Enzymatic activity of BCA was assessed according to the Wilbur-Anderson method [32] with the following modifications. Instead of recording the time that corresponds to a decrease in 2 pH units, the pH was monitored during the analysis, which yielded time courses that denote the activity of the enzyme (as shown in Figure S4). Trizma-SO_4_ buffer (20 mM, pH 8.3) was used and the reaction mixture was kept at under 3 °C during analysis.

### 3.4. Sorption Tests

**Figure 7:**
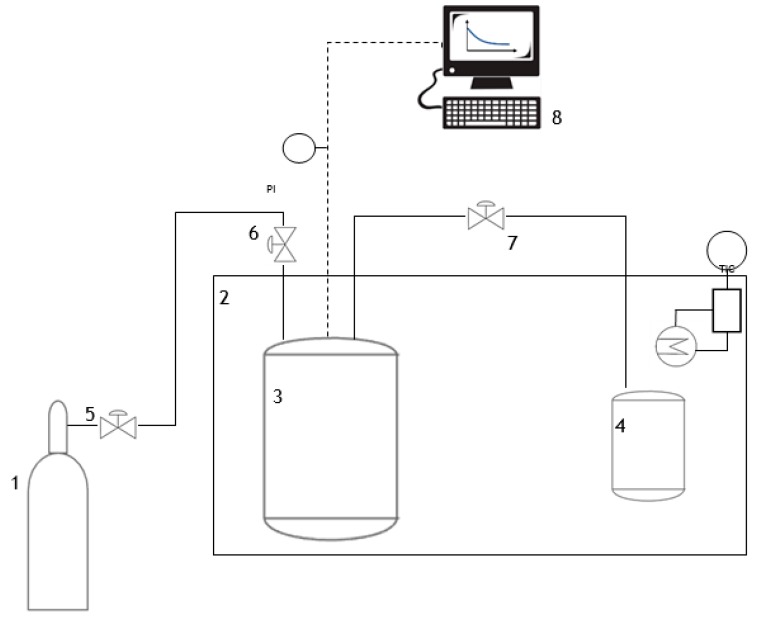
Set up of CO_2_ sorption tests. (1) CO_2_ cylinder, (2) water bath, (3) gas storage cell, (4) absorption cell, (5–7) manual control valves, and (8) computer.

The CO_2_ sorption capacity of the aqueous solutions was assessed in a stainless-steel cell provided with pressure transducers, and located inside a water bath for conductive heat transfer and temperature control (Figure 7). A gas storage cell was used to condition the gas to the desired temperature before absorption. All tests were carried out at 30 °C, with either 2 or 0.5 g of solutions, and an initial pressure of 1.5 bar (absolute). Online measurement of the pressure decay in the headspace was recorded in a computer (1 s interval) and used for the calculation of sorption coefficients (S) and sorption rates, as shown in Equations (1) and (2), respectively. Further details on the experimental procedure were previously reported by our group [33].

(1) $$S_{\mathrm{CO}2}=\frac{\Delta p\cdot V\cdot M_{w}}{R\cdot T\cdot m\cdot 10^{3}}$$

(2) $$r_{\mathrm{CO}2}=\frac{\Delta p\cdot V\cdot 10^{9}}{R\cdot T\cdot \Delta t}$$

where S_CO2_ is the sorption coefficient (g_CO2_ kg^−1^_solution_), Δp is the pressure difference along time (bar), V is the headspace volume (cm^3^), M_w_ is CO_2_ molecular weight (44 g mol^−1^), R is the universal gas constant (83.14 bar cm^3^ mol^−1^ K^−1^), T is the absolute temperature (K), m is the sample mass (g), r_CO2_ is the sorption rate (nmol s^−1^), and Δt is the time span (s). r_CO2_ was determined from pressure data within the initial 15 min of the test after smoothing treatment to reduce noise from the readings. R^2^ of at least 0.94 was considered as the threshold for data reporting. 

## 4. Conclusions

Structural studies on the BCA under industrially relevant aqueous conditions (alkaline pH) for CO_2_ capture suggest that the enzyme changes its conformation at temperatures of 61–72 °C, which are lower than typical stripping stages, which evidences the need to adopt stabilization approaches for further implementation of processes using this enzyme. Regarding the pH, BCA showed better performance for CO_2_ absorption at pH 10 (this is the pH where the enzyme presents higher stability, which is demonstrated by the CD results) with a more marked improvement on the kinetic (r_CO2_) than equilibrium (S_CO2_) variables, which results in the acceleration of the CO_2_ capture rate up to 43%. The biocatalytic route of CO_2_ sequestration from industrial streams presents high technical potential and should be further investigated by mostly focusing on kinetic improvements aimed at continuous processes.

## Figures and Tables

**Table 1:** Mineral water (MW) composition.

Ion	Concentration (mg L^−1^)
Ca^+2^	0.386 ± 0.031
K^+^	2.253 ± 0.053
Mg^+2^	0.055 ± 0.007
Na^+^	8.871 ± 0.267
Al^+3^	n.d.
Fe^+2^	n.d.
Cl^−^	38.976 ± 0.071
NO_2_^−^	4.804 ± 0.200
SO_4_^−2^	54.911 ± 0.205

n.d.: not detected.

**Table 2:** Melting temperature of BCA at different pH conditions. R^2^ value corresponds to the fitted CD curve to the two-state model.

pH	T_m_ (°C)	R^2^
9	72.82 ± 0.29	0.9783
10	70.45 ± 0.32	0.9738
11	61.78 ± 0.36	0.9707

**Table 3:** pH and conductivity changes at different initial pH conditions during CO_2_ absorption (unbuffered media). Mean standard deviation is 3% in both responses.

Initial pH	Enzyme Addition	pH Drop	Conductivity Change (μS cm^−1^)
9	no	2.78	+124
	yes	2.37	+17
10	no	3.42	−70
	yes	3.45	+78
11	no	4.23	−533
	yes	4.26	−488

**Table 4:** The effect of enzyme concentration on the specific CO_2_ absorption. Blank data (no BCA added) refers to tests in mineral water, whereas data with BCA discount the water contribution. The mean standard deviation is 4%.

Enzyme Concentration (mg g^−1^)	Net Specific CO_2_ Absorption (mol_CO2_ mol^−1^_specie_)
Blank	0.00180
0.2	8014
0.5	5071
2	1005
3	761

## Supplementary Materials

Supplementary materials can be found at https://www.mdpi.com/1422-0067/21/8/2918/s1.
