# Supplementary material for: Biocatalytic CO2 Absorption and Structural Studies of Carbonic Anhydrase under Industrially-Relevant Conditions
Source: Int J Mol Sci. 2020 Apr 22;21(8):2918. doi: 10.3390/ijms21082918 (PMC7215295; doi:10.3390/ijms21082918)
Supplement: Supplementary file 1 [file ijms-21-02918-s001.pdf]

Supplementary material

Biocatalytic CO_2 absorption and structural studies of carbonic anhydrase under industrially-relevant conditions

Aline M. de Castro[1,2,*], Elisabete Ferreira[3], Carla Portugal[1], Luisa A. Neves[1] and João G. Crespo[1,*]

[1] LAQV, REQUIMTE, Departamento de Química, Faculdade de Ciências e Tecnologia, Universidade NOVA de Lisboa, 2829-516, Caparica, Portugal

[2] Biotechnology Division, Research and Development Center, PETROBRAS, Av. Horácio Macedo, 950. Ilha do Fundão, Rio de Janeiro, 21941-915, Brazil.

[3]UCIBIO, REQUIMTE, Faculdade de Ciências e Tecnologia, Universidade NOVA de Lisboa, Campus de Caparica, 2829-516, Caparica, Portugal

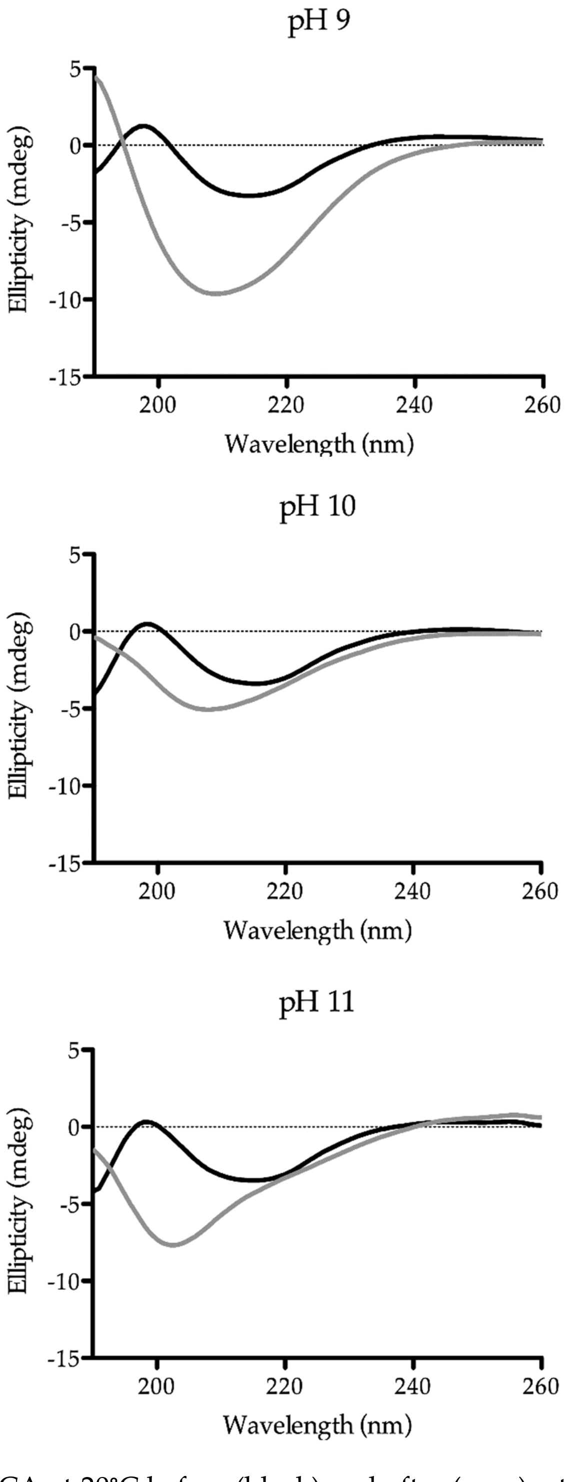

Figure S1: CD spectra of BCA at 20°C before (black) and after (grey) a temperature ramp (20-94 °C) under different pH conditions in mineral water.

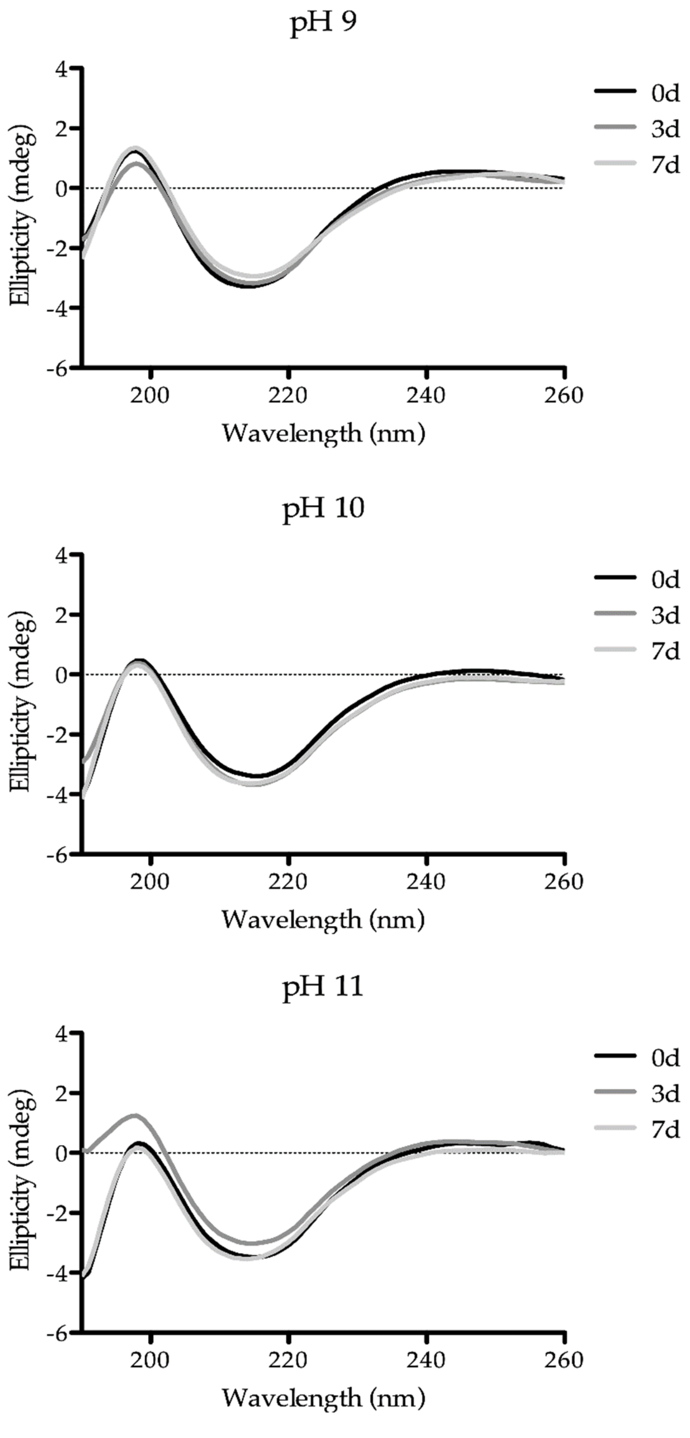

Figure S2: CD spectra of BCA during incubation at 30 °C under different pH conditions in mineral water.

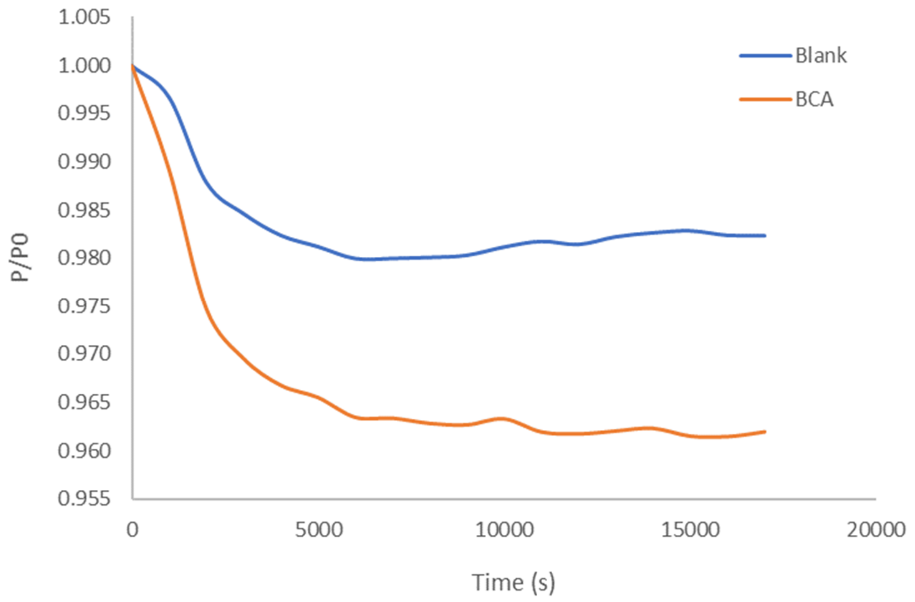

Figure S3: Time courses of pressure decay (normalized to the initial pressure – P_0) as a function of time, during CO_2 sorption tests under pH 10. Raw data are plotted after using a smoothing tool, to minimize noise.

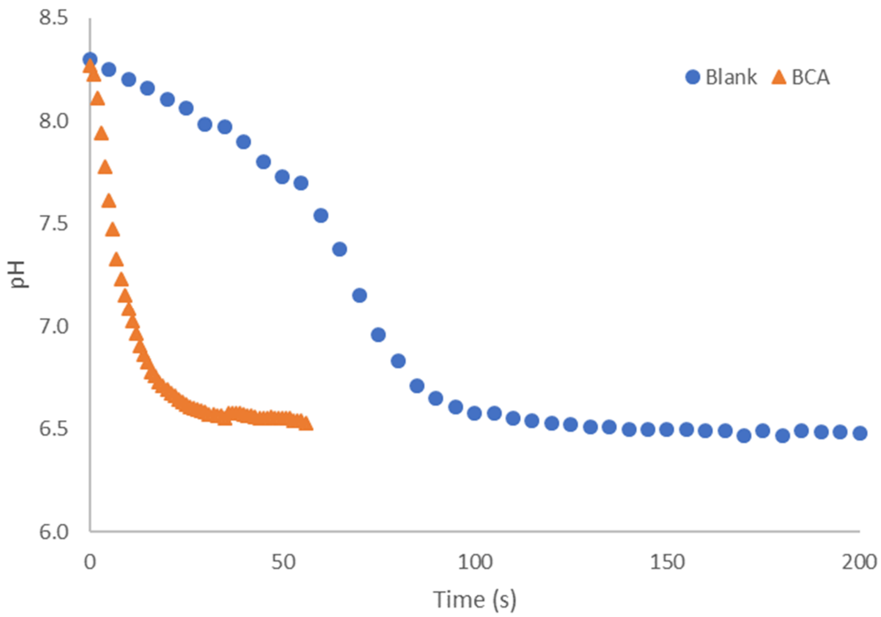

Figure S4: Time courses of pH decrease during determination of the enzymatic activity of BCA, according to Wilbur-Anderson method [1].

Reference

1. Wilbur, K.M.; Anderson, N.G. Electrometric and colorimetric determination of carbonic anhydrase. *J. Biol. Chem*, **1948**, *176*, 147-154.
